# Emerging Roles for the Influenza A Virus Nuclear Export Protein (NEP)

**DOI:** 10.1371/journal.ppat.1003019

**Published:** 2012-12-06

**Authors:** Duncan Paterson, Ervin Fodor

**Affiliations:** Sir William Dunn School of Pathology, University of Oxford, Oxford, United Kingdom; University of Alberta, Canada

## Abstract

Influenza virus is a major human and animal pathogen causing seasonal epidemics and occasional pandemics in the human population that are associated with significant morbidity and mortality. Influenza A virus, a member of the orthomyxovirus family, contains an RNA genome with a coding capacity for a limited number of proteins. In addition to ensuring the structural integrity of virions, these viral proteins facilitate the replication of virus in the host cell. Consequently, viral proteins often evolve to perform multiple functions, the influenza A virus nuclear export protein (NEP) (also referred to as non-structural protein 2, or NS2) being an emerging example. NEP was originally implicated in mediating the nuclear export of viral ribonucleoprotein (RNP) complexes, which are synthesized in the infected cell nucleus and are assembled into progeny virions at the cell membrane. However, since then, new and unexpected roles for NEP during the influenza virus life cycle have started to emerge. These recent studies have shown NEP to be involved in regulating the accumulation of viral genomic vRNA and antigenomic cRNA as well as viral mRNA synthesized by the viral RNA-dependent RNA polymerase. Subsequently, this regulation of viral RNA transcription and replication by NEP was shown to be an important factor in the adaptation of highly pathogenic avian H5N1 influenza viruses to the mammalian host. Unexpectedly, NEP has also been implicated in recruiting a cellular ATPase to the cell membrane to aid the efficient release of budding virions. Accordingly, NEP is proposed to play multiple biologically important roles during the influenza virus life cycle.

Influenza viruses are a major contributor to disease and death in humans, and with their ability to cause yearly epidemics and occasional pandemics, they represent a considerable burden to healthcare systems globally. Influenza viruses are unpredictable, and novel strains, against which there is little or no preexisting immunity in the human population, can arise at any time. Currently the H5N1, H7N7, and H9N2 subtypes are considered to be particular threats, as these are prevalent in birds and can infect humans directly [1]. Although these viruses lack the ability to transmit between humans, they can cause severe disease and death. The concern is that these viruses, through adaptation or reassortment with other influenza virus subtypes, could gain the ability to easily transmit between humans and lead to a new pandemic.

In order to initiate infection, the viral genome composed of eight single-stranded, negative-sense RNA segments must be introduced into a host cell. The viral genomic segments reside within the virion as viral ribonucleoprotein (vRNP) complexes bound to a heterotrimeric RNA-dependent RNA polymerase and the viral nucleoprotein (NP) [2]. Fusion of the viral membrane with the endosomal membrane results in uncoating of the virus and the release of vRNP complexes into the cell cytoplasm, where they are actively imported to the nucleus. Once in the nucleus, the vRNPs are transcribed by the viral polymerase, producing mRNA and a positive-sense cRNA replication intermediate, which is in turn used as a template for vRNA synthesis [2]–[5]. The viral RNA polymerase plays a central role in influenza virus replication and is known to undergo adaptive changes when influenza virus transmits between species. These adaptive changes are likely to facilitate the interaction of the transcriptional machinery with cellular factors involved in the intracellular trafficking and assembly of its components as well as modulate its catalytic activities.

During adaptation to replicate in new host species, influenza viruses must overcome the limited coding potential of a 15 kb genome. To this end, influenza viruses employ several strategies to increase genomic coding capacity, the most significant of which is encoding multiple polypeptides on the same genome segment. In this respect, unspliced and spliced mRNA transcripts of segments 7 and 8 are translated into distinct proteins [6], [7]. Splicing of the precursor viral mRNA is mediated by host cell splicing factors that recognise the conserved intron/exon boundaries on segment 7 and 8 mRNA [8], [9]. In addition to posttranscriptional processing, three viral polypeptides are produced by distinct alternative translation strategies. Intriguingly, two of these polypeptides are significant virulence factors originating from segments 2 and 3 that ordinarily encode the viral polymerase subunits PB1 and PA. The first of these, PB1-F2, is translated from an alternative reading frame within segment 2 encoding an 87–90 amino acid polypeptide and has multiple functions including apoptosis induction [10], regulating the cellular interferon response [11] and modulating host susceptibility to bacterial superinfection [12]. In addition to PB1-F2, segment 2 encodes a product from an in-frame downstream initiation site, producing an N-terminally truncated version of PB1 called N40 [13]. Although there is as yet no defined role for N40 in viral replication, there appears to be a degree of interplay between the translation strategies of PB1-F2 and N40 that may result in the modulation of virus-induced pathogenesis [14], [15]. In contrast to products derived from segment 2, the recently described PA-X polypeptide is the product of ribosomal frameshifting during the translation of segment 3 mRNA [16]. This results in a polypeptide with an N-terminus corresponding to the PA endonuclease domain that plays a role in virus-induced host shut-off and affects the virulence of the 1918 H1N1 pandemic virus.

In addition to extending the coding capacity of the viral genome by differential transcription and translation, influenza viruses also encode proteins that perform more than one function during virus infection. The best studied viral protein in this regard is the nonessential virulence factor NS1. This remarkably multifunctional protein has been shown to inhibit both interferon production and the activity of several interferon-induced genes, as well as inhibit the processing and nuclear export of host mRNA (reviewed in [17]).

In addition to NS1, viral segment 8 encodes a 121 amino acid polypeptide from a spliced form of the segment mRNA transcript [6]. This small protein was originally thought to have no structural function within the virion, leading to its designation as nonstructural protein 2 (NS2) [7], [18]. Subsequently, it was noted that small amounts of NS2 protein were present in virions where it may interact with the viral matrix protein M1 [19]–[21]. NS2 was later implicated in mediating the export of vRNPs from the host cell nucleus, thereby ensuring that the viral genomic segments are available for packaging into daughter virions on the cellular periphery [22]. This led to the proposal that the NS2 protein be renamed as the nuclear export protein (NEP).

Recent studies have suggested that NEP may have more than one function during the influenza virus replication cycle (Figure 1). In addition to nuclear export of vRNPs, it has been demonstrated that NEP contributes to the viral budding process through interaction with a cellular ATPase [23]. Furthermore, studies have demonstrated that NEP is capable of regulating the accumulation of viral RNA species, potentially leading to a switch from viral transcription during early viral replication to favour the production of genomic vRNPs [24], [25]. In this regard, there is substantial evidence that mutations in NEP capable of increasing viral RNA replication are able to confer a significant replicative advantage during mammalian adaptation of a highly pathogenic avian influenza virus [25]. Consequently, NEP appears to perform several distinct, biologically important functions during influenza virus replication.

**Figure 1:**
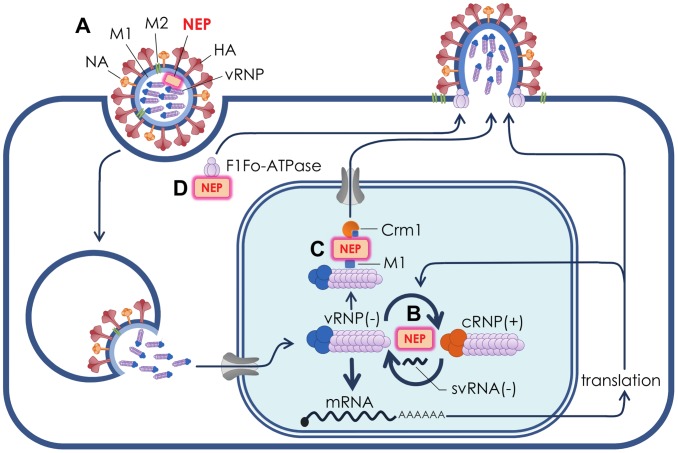
Roles of NEP in influenza virus replication. (A) The influenza virion is an enveloped particle with an outer surface dominated by the receptor-binding protein haemagglutanin (HA) and the sialic acid-cleaving neuraminidase (NA), as well as small amounts of the M2 ion channel. The viral matrix protein (M1) is situated beneath the lipid envelope. The segmented virus genome is packaged in the form of viral ribonucleoproteins (vRNPs) that comprise the three viral polymerase subunits PA, PB1, and PB2 and multiple copies of the nucleoprotein (NP) bound to the viral genomic RNA. NEP was first thought to be nonstructural in function, however it is now recognised that NEP is resident within influenza virions where it may interact with M1. (B) NEP stimulates the synthesis of the viral cRNP replication intermediate that is proposed to result in the increased production of vRNPs late in infection for packaging into progeny virions. In order to achieve this, NEP may act in concert with recently described small viral RNAs (svRNAs). (C) NEP acts as an adaptor protein to mediate the export of vRNPs from the nucleus for packaging into progeny virions at the cell periphery. NEP mediates nuclear export by interacting with the cellular nuclear export protein Crm1 and the viral M1 protein, which is in turn bound to vRNPs. (D) NEP has been shown to recruit the F1Fo ATPase that is involved in the budding of progeny virions.

## NEP Organisation and Structure

**Figure 2:**
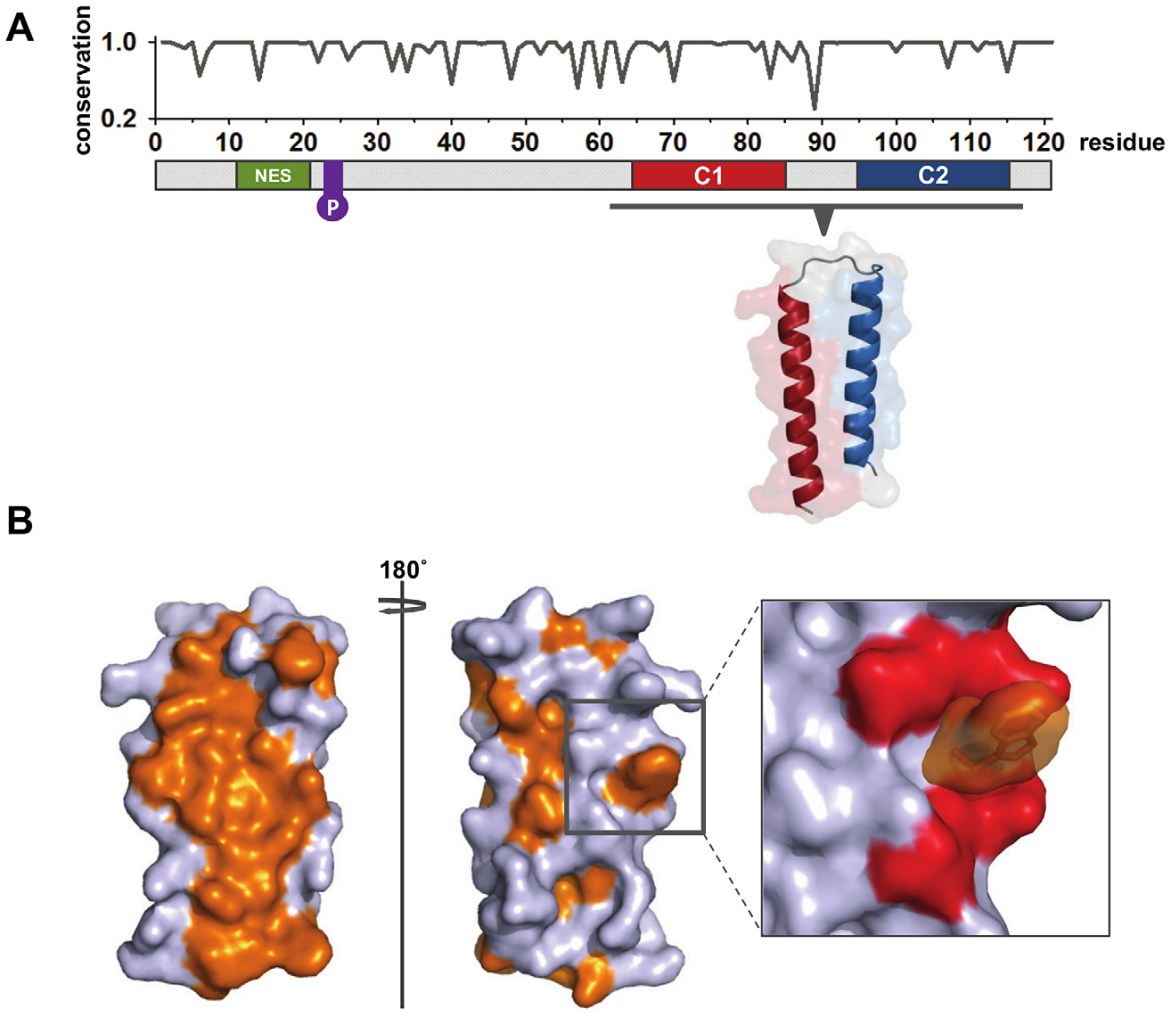
Organisation and structure of influenza virus A NEP. (A) A schematic representation of NEP including amino acid conservation at each position marked graphically above. Conservation at each amino acid position was calculated using Bioedit (Ibis Biosciences) from alignments of 16065 full-length influenza A NEP sequences obtained from http://www.fludb.org (accessed March 14, 2012). Alignments of all sequences were conducted using MAFFT [76]. The N-terminal nuclear export signal (NES) enables binding of NEP to the cellular β-importin Crm1. A highly conserved serine-rich motif at positions 23–25 (purple) can be phosphorylated during virus infection. The C-terminal α-helices interact along their lengths to form an almost perfectly anti-parallel hairpin, the structure of which has been solved (PDB:1pd3; [26]). (B) Hydrophobic residues on the NEP C-terminal domain are depicted in orange. The opposing hydrophobic and hydrophilic faces result in the NEP C-terminal domain forming an amphipathic molecule. In full-length NEP the N-terminal domain is proposed to pack against the hydrophobic face, effectively burying it. The putative M1-binding tryptophan residue (W78, orange) projects prominently from within a ring of glutamate residues (red) on the hydrophilic face of the NEP C-terminal domain.

NEP can be divided into a protease-sensitive N-terminal domain (amino acids 1–53) and a protease-resistant C-terminal domain (amino acids 54–121), the crystal structure of which has been solved (Figure 2A) [26]. Although there is as yet no structural information available for the N-terminal domain, there is considerable evidence for a nuclear export sequence (NES) located between residues 12 and 21 [22], [27]. The NES is proposed to interact with the cellular nuclear export protein Crm1 and is unusual in the sense that three of the five critical hydrophobic residues are methionines rather than the canonical leucine [27], [28]. Although the functional importance is as yet unknown, NEP is phosphorylated during the influenza replication cycle [19]. In this regard, the phosphorylation of a highly conserved serine-rich motif (S23, S24, and S25) proximal to the NES has recently been demonstrated in virion-associated NEP [29], [30]. In addition to phosphorylation, NEP has been identified as a target for in vitro sumoylation; however, it is unclear whether NEP is sumoylated during virus replication or indeed what function this could serve [31].

The highly structured C-terminal domain consists of two α-helices C1 (amino acids 64–85) and C2 (amino acids 94–115) that are connected by a short interhelical turn. The two α-helices are comparable in length and interact extensively, forming an almost perfectly antiparallel hairpin (Figure 2A). The hairpin conformation results in the C-terminal domain being amphipathic in character, with the opposing external faces predominantly displaying hydrophobic and hydrophilic functional groups, respectively (Figure 2B). It is not known if the N-terminal domain interacts with the C-terminal α-helices, however it has been postulated that the N-terminal domain effectively buries the hydrophobic face of the C-terminal hairpin [26]. In contrast, the opposing hydrophilic face of NEP appears to be surface-exposed and displays a prominent hydrophobic tryptophan residue (W78) in the centre of a glutamate cluster (Figure 2B). It has been demonstrated that W78 is required for binding to the viral M1 protein in an interaction that is proposed to be important for vRNP nuclear export [26]. As a whole NEP amino acid identity is highly conserved across all sequenced influenza A strains (93.4%) (Figure 2A). Interestingly, the most conserved of the known NEP structural features is the C2 α-helix, which shows 96.3% conservation across all full-length influenza A NEP sequences.

## Role of NEP in vRNP Export

During influenza virus infection, incorporation of newly synthesised vRNPs into progeny virions is dependent on the export of vRNPs from the host cell nucleus. In this regard, nuclear export of vRNPs appears to be predominantly dependent on the cellular β-importin protein Crm1, which along with its cofactor RanGTP recognises a structurally conserved hydrophobic NES [32]–[35]. Accordingly, influenza virus-infected cells treated with leptomycin B, a potent inhibitor of the Crm1 pathway, show intranuclear retention of vRNPs [32], [33], [36]. Crm1-mediated nuclear export of viral nucleoprotein complexes has been extensively studied in HIV-1 infection where unspliced viral mRNA is exported from the nucleus bound to the Crm1 export complex by way of the NES-containing viral mRNA-binding protein Rev [37]–[39]. Interestingly, the HIV-1 mRNA nuclear export pathway can be reconstituted when the Rev NES is replaced with the influenza virus NEP NES [22]. Moreover, the introduction of anti-NEP antibodies into influenza virus-infected cells was shown to block vRNP export [22], a phenotype that is shared with nonviable recombinant viruses that do not encode NEP [28]. These findings, along with the observation that nuclear localisation of NEP is essential for productive infection [40]–[42], implicate NEP as a key mediator in vRNP nuclear export.

**Figure 3:**
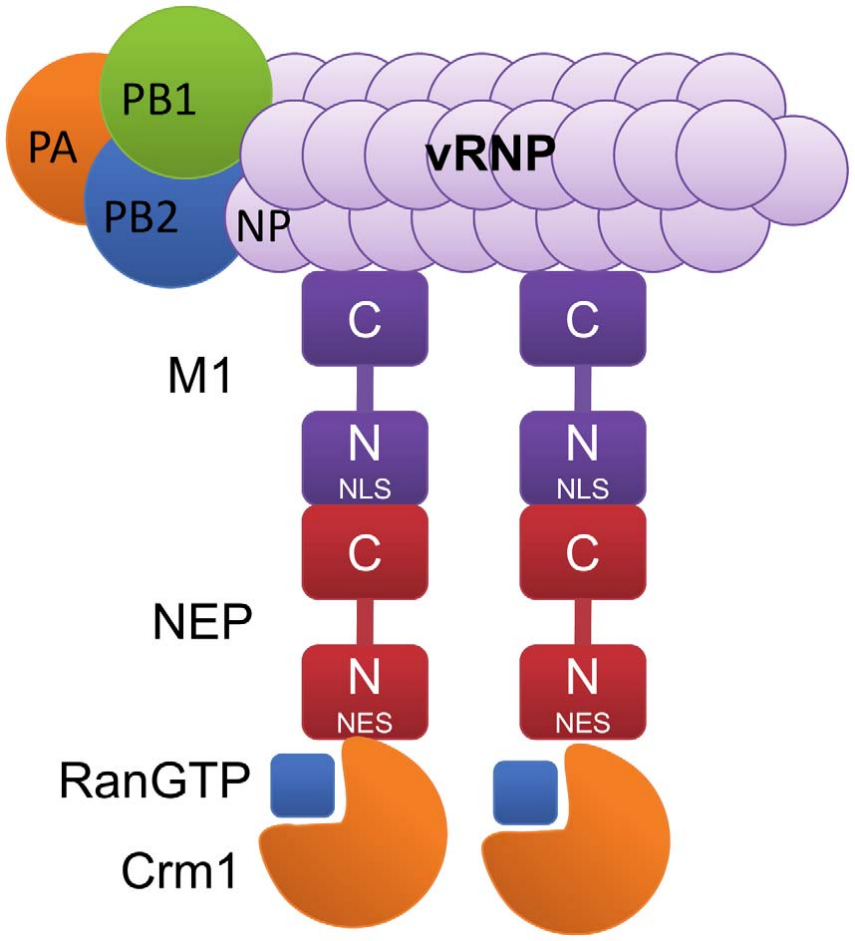
The daisy chain model for NEP-mediated nuclear export of influenza virus vRNPs. The β-importin Crm1 mediates export of the vRNP complex by binding to the N-terminal domain of NEP, as well as to its cofactor, the small GTPase Ran. The C-terminus of NEP binds to the nuclear localisation signal (NLS) on the N-terminal domain of the viral matrix protein M1. The C-terminus of M1 in turn binds strongly to the vRNP through interaction with NP (grey).

This evidence suggests that NEP serves a similar function to the HIV-1 Rev protein by acting as an adapter between vRNPs and the Crm1 export machinery. In this model for vRNP nuclear export (Figure 3), Crm1 recognises the NES on the N-terminus of NEP but binds with RanGTP to an unknown site distinct from the NES [28]. The C-terminal hairpin of NEP in turn associates with the N-terminal nuclear localisation signal (NLS) of the viral matrix protein M1 [20], [21], [26], [43], and the M1 protein binds to the vRNP through a C-terminal interaction with NP [44]. However, it should be noted that the N-terminus and the N-M domain of M1 have also been implicated in NP binding [45], [46]. These interactions have led to the description of the vRNP nuclear export complex as a daisy chain consisting of Crm1, NEP, M1, and vRNP. A recent study has alluded to a further function for NEP after the vRNP-nuclear export complex has exited the nucleus. In this regard, it was demonstrated that the NEP-binding cellular ATPase F1Fo localises at the bottom edge of budding virions and is critical for efficient influenza virus egress [23]. Although the role of NEP in this process is unclear, it is conceivable that F1Fo is recruited by vRNP-bound NEP in the cytoplasm prior to viral genome packaging.

Although a plausible model for vRNP nuclear export has been demonstrated, several studies have called into question the role of NEP as the principal arbiter in this process. In this respect, it has been repeatedly noted that NEP and M1 do not colocalise with vRNPs, as would be expected if this was the sole mechanism for exporting viral genome segments from the nucleus [32], [33]. In addition, disruption of the Crm1 pathway following treatment with leptomycin B does not alter the localisation of M1 or NEP but does cause accumulation of NP at the nuclear periphery [32]. Similarly, overexpression of Crm1 shows no effect on the localisation of NEP [32]. It is important to note that these studies rely heavily on fluorescence microscopy, and it is possible that small differences in NEP and M1 localisation have gone undetected. However, a potential role for NP in vRNP nuclear export should not be dismissed. NP has been shown to actively accumulate in lipid rafts at the apical cell membrane in the absence of other viral proteins, a role that has been proposed to govern the polarity of viral budding [47]. Indeed, NP is a structural component of vRNPs and has been shown to contain three functional nuclear export signals, one of which is Crm1-dependent [26], [32], [48]. Interestingly, the interaction between NP and Crm1 has been proposed to facilitate wild-type levels of vRNP nuclear export in cells infected with a mutant virus that expresses greatly reduced levels of NEP [32]. Incidentally, even in cells infected with a virus expressing significantly decreased levels of NEP, no co-localisation was observed between NEP and Crm1 [32]. Further studies have found that in addition to NEP and NP, M1 may also be capable of mediating nuclear export of vRNPs [42]. In this case, nuclear retention of vRNPs could be overcome by exogenously expressed M1 in cells blocked for the expression of late viral proteins including NEP [42]. In agreement with this finding, M1 has recently been shown to contain an NES capable of mediating Crm1-independent nuclear export, the mutagenesis of which results in a significant decrease in viral titre [49].

In addition to the extensively studied roles of influenza virus-encoded proteins in vRNP nuclear export, several cellular factors have been proposed to contribute to this process. Perhaps the most interesting of these findings is that inhibition of the (mitogen-activated protein kinase) MAPK signalling cascade leads to impaired nuclear export of vRNPs by interfering directly with NEP-mediated export [50]. Further studies present evidence that MAPK signalling is activated by protein kinase Cα (PKCα) late in influenza virus infection as a result of HA accumulation on lipid rafts at the cell membrane [51]. This suggests that membrane accumulation of HA has the potential to act as a switch that activates MAPK signalling, thereby directing the nuclear export of vRNPs to the cell membrane for packaging into progeny virions. It is, however, unclear how vRNP nuclear export would be triggered, as NEP is not a direct target of MAPK signalling (i.e., activation of the MAPK cascade does not result in NEP being phosphorylated) [50]. Another cellular factor that has been demonstrated to influence vRNP nuclear export is the apoptotic regulator caspase 3 [52]. In this regard, it has been observed that inhibition of caspase 3 leads to a phenotype similar to that of MAPK signalling inhibition or treatment with leptomycin B, characterised by the nuclear retention of vRNPs. As a result, caspase 3 is proposed to play a role in vRNP nuclear export by increasing the diffusion limit of nuclear pores in a manner that is independent of MAPK signalling and Crm1 [52].

These observations indicate that there is the potential for considerable interplay between viral proteins and several distinct cellular factors during the nuclear export of vRNPs. Consequently, there appears to be significant redundancy in the influenza virus nuclear export pathway.

## Emerging Role for NEP during Transcription and Replication

In addition to the much-studied role of NS2/NEP in vRNP export, several studies have alluded to a novel role for NEP during influenza virus replication. Although a biochemical mechanism has yet to be described, NEP appears to play a significant role in regulating the accumulation of influenza virus mRNA, cRNA, and vRNA. Moreover, this function of NEP has been demonstrated to play a critical role in the adaptation of some avian H5N1 influenza viruses to efficient replication in mammalian cells [25].

The first study to conclude that NEP could influence events other than the export of vRNPs demonstrated that a single point mutation, I32T, results in highly efficient production of defective interfering (DI) particles lacking an intact *PA* gene [53], [54]. In viruses containing the mutated NEP, replication of the full-length PA segment vRNA was shown to be suppressed during cRNA synthesis, whereas replication of the DI vRNA was enhanced [53]. While the evidence for the formation of DI particles during natural infection is controversial [55], [56], it is evident that NEP could play a role during vRNA genome replication and mutations affecting this function may lead to the replication of short, subgenomic RNA species.

Although the role of NEP in the production of aberrant genomic replication products is intriguing, it sheds little light on the role of NEP during virus replication. Subsequently, however, NEP was demonstrated to inhibit reporter gene expression in a dose-dependent manner using an influenza virus mini-replicon system [57]. Moreover, it was demonstrated that the NEP concentrations required to significantly inhibit reporter gene expression could physiologically be reached within an infected cell, indicating that this is unlikely to be an artefact of NEP overexpression [57]. Further experiments conducted using intracellular vRNP reconstitution assays have corroborated the evidence that higher levels of NEP expression inhibit the transcription and replication of all viral RNA species [25].

Recent studies have shown that, in addition to inhibiting viral genomic replication and transcription, low concentrations of NEP can stimulate the accumulation of influenza virus vRNA and cRNA [24], [25]. Consequently, it was proposed that NEP plays a critical role in determining the viral mRNA:cRNA ratio within infected cells. Moreover, regulation by NEP occurred independently of viral M1 protein expression and did not require the presence of the NEP NES [24]. This has led to the proposal that the regulatory role of NEP is mechanistically distinct from the vRNP nuclear export pathway. Interestingly, as is the case for interaction with M1 during nuclear export, NEP regulation requires the C-terminal α-helices, although mutation of the putative M1-binding residue (W78) showed no effect on regulatory activity [24]. Furthermore, the ability of NEP to regulate RNP accumulation was conserved between influenza type A and B viruses in a type-specific manner, further alluding to the critical role of NEP-regulated viral RNA transcription during influenza virus replication [24], [57].

**Figure 4:**
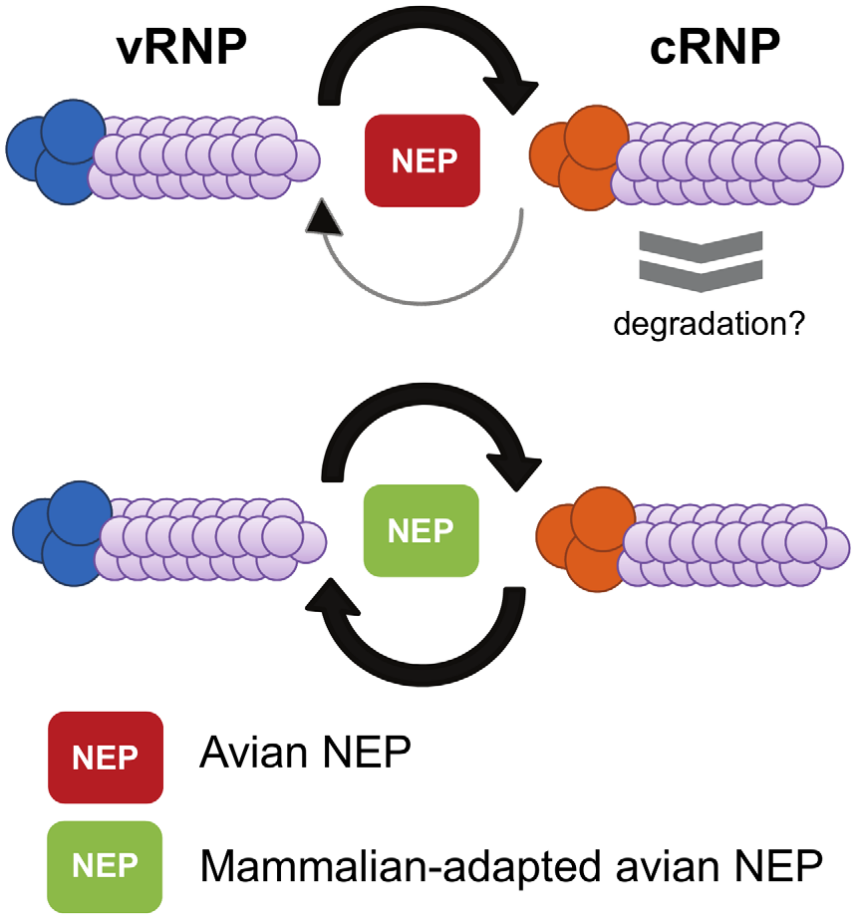
Mutations in NEP are responsible for overcoming host-range restriction. Adaptive mutations in NEP can result in an increase in viral RNA accumulation, thereby allowing avian influenza viruses to overcome host restriction in mammalian cells. Avian influenza viruses show restricted replication in mammalian hosts due to inefficient viral RNA synthesis. This has been proposed to result from decreased cRNP stability. Adaptive mutations in NEP can overcome this restriction by increasing viral RNA accumulation.

Until recently, the biological significance of NEP-mediated regulation of viral RNA accumulation was unknown. However, a recent study has demonstrated that mutations within NEP were responsible for the adaptation of a highly pathogenic avian influenza virus, thereby enabling increased replication in mammalian cells [25]. This is of biological importance as most avian influenza viruses do not replicate efficiently in mammalian cells [58]. One of the factors that leads to this host-range restriction is the reduced activity of avian viral polymerases in mammalian cells [58]–[60]. The reduction in polymerase activity has been ascribed to impaired cRNP synthesis by avian influenza polymerases in mammalian cells [25]. However, restricted avian influenza virus polymerase activity could be overcome by a number of ostensibly nonrelated compensatory point-mutations in NEP, causing an increase in the accumulation of cRNPs (Figure 4) [25]. It is interesting to note that compensatory mutations occurred in both the N- and C-terminal domains of NEP and that NEP has previously been implicated in phylogenetic studies on host-range adaptation [61], [62]. In addition, mutations in NEP capable of enhancing the activity of avian-derived viral polymerases were found in several circulating influenza strains including the pandemic H1N1 strain [25]. Intriguingly, the compensatory function of NEP may hint towards a potential mechanism for NEP regulation of viral RNA accumulation. The C-terminal α-helices of NEP were demonstrated to interact directly with the two basic polymerase subunits, PB1 and PB2 [25]. Both PB1 and PB2 have been implicated in promoter binding [63]–[68], and it is therefore tempting to speculate that NEP binds to the polymerase and acts as a co-factor to stimulate viral genomic replication. Taken together, this and previous studies suggest that NEP may function in switching the role of the viral polymerase from transcription early in infection to the production of genomic vRNPs by up-regulating cRNP synthesis [24], [25].

In a recent study, a novel species of virally encoded RNA was implicated in regulating the switch from viral transcription to replication [69]. These short single-stranded molecules, dubbed small viral RNAs (svRNAs), are 22–27 nucleotides in length and correspond to the 5′ end of each of the eight vRNA segments [69], [70]. During the late stages of viral infection, svRNAs are proposed to regulate vRNA replication, favouring the synthesis of the cRNA replication intermediate over mRNA transcription. It is suggested that svRNA-mediated regulation of vRNA replication is conferred through binding to the viral polymerase, thereby serving as a segment-specific guide capable of augmenting the vRNA promoter so as to favour the synthesis of full-length cRNA over prematurely terminated and polyadenylated mRNA transcripts. Interestingly, expression of only the polymerase subunits, nucleoprotein, and negative-sense template is not sufficient for the generation of svRNAs; however, the addition of NEP to the RNP reconstitution assay leads to svRNA accumulation [69]. Nonetheless, it remains unclear whether the requirement for NEP alludes to a prospective role during an svRNA-mediated switch from transcription to replication, or if the stimulatory activity of NEP on viral genomic replication results in the detectable accumulation of short aborted viral transcripts produced during normal transcription by the influenza virus polymerase.

Although the mechanism for NEP-mediated regulation of transcription and replication requires further elucidation, these studies all suggest a role for NEP during influenza virus infection that is independent of vRNP export. In this respect, it is salient to consider that other negative-sense viruses encode proteins, other than the nucleocapsid components, capable of regulating transcription and replication of their respective viral genomes. In particular, the NS1, NS2, and M2-2 proteins of human respiratory syncytial virus down-regulate viral RNA transcription and replication [71]–[73] and the C protein of Sendai virus specifically inhibits RNA synthesis from the genomic promoter [74]. Likewise, the P protein of parainfluenza virus, which does not possess enzymatic activity but is an essential co-factor for viral polymerase activity, regulates viral mRNA synthesis in a phosphorylation-dependent manner [75]. Accordingly, it is conceivable that influenza virus NEP may share functional similarities with these viral regulatory proteins.

In conclusion, NEP has a well-studied role as an adapter protein during Crm1-mediated export of influenza virus vRNP complexes from the nucleus of infected cells. In addition, recent studies have implicated NEP in controlling the accumulation of viral RNA species within the host cell, in a manner that is distinct from its vRNP export function. Although the mechanism of this novel function remains unelucidated, it is tempting to suggest that NEP is a crucial factor, either independently or along with svRNA, in regulating viral transcription and replication.
